# Impact of the COVID‐19 pandemic on clinical presentation, treatments, and outcomes of new breast cancer patients: A retrospective multicenter cohort study

**DOI:** 10.1002/cam4.6637

**Published:** 2023-11-01

**Authors:** Etienne Guével, Sonia Priou, Guillaume Lamé, Johanna Wassermann, Romain Bey, Catherine Uzan, Gilles Chatellier, Yazid Belkacemi, Xavier Tannier, Sophie Guillerm, Rémi Flicoteaux, Joseph Gligorov, Ariel Cohen, Marc‐Antoine Benderra, Luis Teixeira, Christel Daniel, Barbara Hersant, Christophe Tournigand, Emmanuelle Kempf, the Assistance Publique–Hôpitaux de Paris (AP‐HP) Cancer Group, a Cancer Research Application on Big Data (CRAB) initiative

**Affiliations:** ^1^ Assistance Publique–Hôpitaux de Paris, Innovation and Data, IT Department Paris France; ^2^ CentraleSupélec, Laboratoire Génie Industriel Université Paris‐Saclay Gif‐sur‐Yvette France; ^3^ Assistance Publique–Hôpitaux de Paris, Department of medical oncology, Pitié Salpétrière University Hospital Sorbonne Université Paris France; ^4^ Assistance Publique–Hôpitaux de Paris, Institut Universitaire de cancérologie Sorbonne Université Paris France; ^5^ Assistance Publique–Hôpitaux de Paris, Department of gynecology, Pitié Salpétrière University Hospital Sorbonne Université Paris France; ^6^ Department of medical informatics, Assistance Publique Hôpitaux de Paris, Centre‐Université de Paris (APHP‐CUP) Université Paris CIté Paris France; ^7^ Assistance Publique–Hôpitaux de Paris, Department of Radiation Oncology and Henri Mondor Breast Center, Henri Mondor and Albert Chenevier University Hospital Université Paris Est Créteil Créteil France; ^8^ Sorbonne University Inserm, Université Sorbonne Paris Nord, Laboratoire d'Informatique Médicale et d'Ingénierie des Connaissances pour la e‐Santé, LIMICS Paris France; ^9^ Assistance Publique–Hôpitaux de Paris, Department of radiation therapy, Saint Louis University Hospital Université Paris Cité Créteil France; ^10^ Assistance Publique–Hôpitaux de Paris Department of medical information Paris France; ^11^ Assistance Publique–Hôpitaux de Paris, Department of medical oncology, Tenon University Hospital Sorbonne Université Paris France; ^12^ Assistance Publique–Hôpitaux de Paris, Department of senology, Saint Louis Teaching Hospital Université Paris Cité Paris France; ^13^ Assistance Publique – Hôpitaux de Paris, Department of plastic surgery, Henri Mondor and Albert Chenevier University Hospital Université Paris Est Créteil Créteil France; ^14^ Assistance Publique – Hôpitaux de Paris, Department of medical oncology, Henri Mondor and Albert Chenevier University Hospital Université Paris Est Créteil Créteil France

**Keywords:** COVID‐19, breast neoplasms, early detection of cancer, health services research, quality of health care, routinely collected health data

## Abstract

**Background:**

The SARS CoV‐2 pandemic disrupted healthcare systems. We compared the cancer stage for new breast cancers (BCs) before and during the pandemic.

**Methods:**

We performed a retrospective multicenter cohort study on the data warehouse of Greater Paris University Hospitals (AP‐HP). We identified all female patients newly referred with a BC in 2019 and 2020. We assessed the timeline of their care trajectories, initial tumor stage, and treatment received: BC resection, exclusive systemic therapy, exclusive radiation therapy, or exclusive best supportive care (BSC). We calculated patients' 1‐year overall survival (OS) and compared indicators in 2019 and 2020.

**Results:**

In 2019 and 2020, 2055 and 1988, new BC patients underwent cancer treatment, and during the two lockdowns, the BC diagnoses varied by −18% and by +23% compared to 2019. De novo metastatic tumors (15% and 15%, *p* = 0.95), pTNM and ypTNM distributions of 1332 cases with upfront resection and of 296 cases with neoadjuvant therapy did not differ (*p* = 0.37, *p* = 0.3). The median times from first multidisciplinary meeting and from diagnosis to treatment of 19 days (interquartile 11–39 days) and 35 days (interquartile 22–65 days) did not differ. Access to plastic surgery (15% and 17%, *p* = 0.08) and to treatment categories did not vary: tumor resection (73% and 72%), exclusive systemic therapy (13% and 14%), exclusive radiation therapy (9% and 9%), exclusive BSC (5% and 5%) (*p* = 0.8). Among resected patients, the neoadjuvant therapy rate was lower in 2019 (16%) versus 2020 (20%) (*p* = 0.02). One‐year OS rates were 99.3% versus 98.9% (HR = 0.96; 95% CI, 0.77–1.2), 72.6% versus 76.6% (HR = 1.28; 95% CI, 0.95–1.72), 96.6% versus 97.8% (HR = 1.09; 95% CI, 0.61–1.94), and 15.5% versus 15.1% (HR = 0.99; 95% CI, 0.72–1.37), in the treatment groups.

**Conclusions:**

Despite a decrease in the number of new BCs, there was no tumor stage shift, and OS did not vary.

## Graphical abstract

After a decrease during the first lockdown, the overall number of new breast cancers was comparable in 2020 and 2019. The rate of metastatic stages was similar: 14.8% de novo metastatic tumors in 2020 versus 15.3% in 2019 (*p* = 0.95). There was no local stage shift: pTNM and ypTNM distributions were stable across 1628 resected cases (*p* > 0.3). Access to curative and palliative treatments including plastic surgery (17% and 15%) remained stable (*p* = 0.8). No significant variations in delays of initial care management and patients' overall survival.

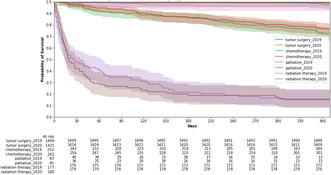

## 1 INTRODUCTION

At the start of the European SARS CoV‐2 pandemic in 2020, most healthcare systems were heavily impacted. Governments and public health authorities implemented national lockdowns and social distancing policies to reduce contamination. These disruptions may have affected the care trajectories of cancer patients across the world.^1^ Indeed, the number of newly referred solid cancer patients dropped during the first waves of the pandemic, and it is not clear whether this drop in diagnoses was compensated later.^2^ In the specific case of breast cancer (BC), which accounts for more than 30% of female cancers, national BC screening programs were interrupted in many countries at some point in 2020.^3^ A meta‐analysis showed that the number of patients screened for BC dropped by 40% at the beginning of the pandemic compared to the pre‐pandemic period, a result confirmed by other systematic reviews.^4^, ^5^, ^6^ Recent nationwide studies showed that national BC screening programs may have reached back to pre‐pandemic levels in the United Kingdom, but not in the United States.^7^, ^8^ As a result of these disruptions, modelers have anticipated a worldwide increase in BC mortality by 2030 because of delays in diagnosis and treatment.^9^, ^10^ However, real‐world evaluations of the impact of the outbreak on care trajectories and outcomes of new BC cases remain scarce, include a low number of patients, and/or lack mid‐term follow‐up of patients' clinical outcomes.^10^, ^11^, ^12^, ^13^, ^14^, ^15^

In France, national guidelines recommended to postpone BC screening and reconstruction surgeries during the initial outbreak, and to prioritize surgical and radiation therapies according to the severity of BC cases.^16^, ^17^ The National Cancer Institute reported a drop of 10% in the annual number of mammograms (−492,500 procedures) and a 1.4%–1.7% drop in BC surgery between 2019 and 2020.^18^, ^19^ At a regional level, the impact of the pandemic on initial tumor stages and care pathways varied. In Burgundy, the authors reported no impact on BC initial presentation or times to anticancer treatment in 2020 compared to 2019.^20^ In South West France, investigators reported less new BC cases, diagnosed with larger tumors.^21^ In Paris region, a comprehensive cancer center reported no impact of the pandemic on clinical presentation or time to treatment, despite a 42% decrease in the number of initial visits for recently diagnosed BC during the first national lockdown (March–May 2020).^22^

The aim of this study was to assess the impact of the SARS CoV‐2 pandemic on tumor stage at diagnosis, anticancer upfront treatments, and 1‐year overall survival (OS) of newly referred BC cases, in the Greater Paris area, before, during, and after the outbreak of the SARS CoV‐2 pandemic in early 2020.

## 2 METHODS

We performed a retrospective multicenter cohort study on the Clinical Data Warehouse (CDW) of Greater Paris University Hospitals (Assistance Publique–Hôpitaux de Paris, AP‐HP), following the REporting of studies Conducted using Observational Routinely‐collected Data (RECORD) extension of the Strengthening the Reporting of Observational studies in Epidemiology (STROBE) guidelines.^2^, ^26^ AP‐HP's CDW was initiated in 2015 to help monitor the quality of care and to promote secondary use of electronic health records (EHR) for research. It contains routinely collected data for 11.4 million patients from 2015 onwards,^23^ covering 39 teaching hospitals in Paris area. The data include patient demographics, claims data, vital status, lab and imaging results, images, and clinical notes.^24^ During the SARS CoV‐2 pandemic, AP‐HP's CDW adapted its procedures to support managers, researchers, and caregivers efficiently.^25^

We identified patients and care events using the CDW's claims database, which contains two types of structured data: diagnoses, based on the International Classification of Diseases (ICD, 10th edition), and medical procedures, based on the French Common Classification of Medical Procedures (CCAM, 11th edition). We identified female patients newly referred between January 1, 2019, and December 31, 2020, to one of the 28 AP‐HP teaching hospitals for which clinical data was available since January 2017. We included patients with (1) an ICD‐10 code C50 or D05 (principal or related diagnosis), not previously registered in the previous 18 months, and (2) no other primary cancer code. We considered that patients had received their BC diagnosis at AP‐HP if one of the CCAM codes related to breast biopsy was present (Table S1). Male patients were excluded from the study.

For the analysis of treatment strategies, we classified patients into four mutually exclusive categories: BC surgery (regardless of any perioperative cancer treatment, using CCAM codes in Table S1), exclusive parenteral systemic anticancer therapy (chemotherapy ICD‐10 Z511, regardless of radiation therapy), exclusive radiation therapy (no chemotherapy, no BC surgery), and exclusive best supportive care (BSC) (ICD‐10 Z515 with no BC anticancer treatment). Patients with no treatment code at AP‐HP were excluded from this analysis. We calculated the 3‐month moving average (average of the month of interest with the previous and following months) of treatment distribution, within the 18 months after the first BC diagnosis code. We compared the proportions of patients who received neo‐adjuvant chemoradiation therapy (ICD‐10 code Z5101) or systemic neo‐adjuvant chemotherapy (ICD‐10 Z511) in 2019 and 2020. We defined elderly patients as those over the age of 70. We compared the rate of BC surgery for these patients during the lockdown periods (March 17–May 11, 2020, October 30–December 15, 2020) to the rates before and after those lockdowns.

We assessed the time between a patient's first multidisciplinary team meeting (MDM) and the start of their BC treatment (the date of BC diagnosis was not exploitable because most BC screening and diagnostic procedures are performed outside the AP‐HP teaching hospitals). We obtained the date of MDM from the structured metadata attached to each MDM report, and we identified the treatment date using claims data. Two separate analyses were conducted depending on which event happened first. For patients who had cancer diagnosis performed in one of AP‐HP's hospitals, we also calculated the median delay between the issue of the pretreatment pathology report and the date of the first treatment.

We used rule‐based natural language processing algorithms to identify the initial tumor stage using (1) baseline PET/CT‐scan and CT‐scan reports (i.e., those coded between 90 days before and 45 days after the diagnosis date) and (2) the first postoperative pathology report for resected tumors (details on the development and validation of these algorithms are provided in the Data S1).^27^ The metastatic tumor stage was identified in the conclusion sections of baseline PET/CT‐scan and CT‐scan reports. We classified resected tumors by ypTNM and pTNM stage (8th WHO TNM classification) according to the risk of relapse, with low‐ and high‐risk defined as (y)pTxN0 and (y)pTxN1‐2, respectively. The “p” in pTNM/ypTNM denotes that the stage was established based on a pathology report, and the “y” in ypTNM indicates that the patient received neoadjuvant treatment.^28^

We analyzed BC patients' OS using daily deaths data obtained by the routine chaining of AP‐HP's CDW and the National Death Registry (NDR) supervised by the French National Institute of Statistics and Economic Studies (INSEE). A patient's OS was defined as the time between the first occurrence of an ICD‐10BC code and the date of the patient's death. Living patients were censored at the date of the last NDR update in AP‐HP's CDW (June 2022). We plotted survival curves plotted using the Kaplan–Meier method. We compared the OS of BC patients referred in 2019 and 2020 (overall, per age class, and per anticancer treatment category). To do so, we estimated hazard ratios (HRs) and their 95% confidence intervals (CIs) through a Cox time‐varying proportional hazard model, with age as a constant covariate and SARS CoV‐2 infection as a time‐varying covariate. We identified SARS CoV‐2 infections by looking for PCR results, positive serologic tests, or the presence of one of the U071 ICD‐10 codes on the year following the date of the BC diagnosis.

We used the chi‐squared test to compare categorical variables and Mann–Whitney's *U* test for quantitative variables. Final data extraction was performed on December 5, 2022. Statistical significance was set at *p* < 0.05. All calculations were performed using Python release 3.7 (www.python.org).

The French Data Protection Agency (CNIL, Commission Nationale de l'Informatique et des Libertés) authorized the constitution of AP‐HP's CDW on January 19, 2017 (approval no. 19800120). In a single commitment to the CNIL, AP‐HP declared that its CDW complied with the French national reference methodology MR‐004 for processing personal data for the purposes of research of public interest nature and that does not involve the human person, which includes research reusing existing data. The study reported in this article was approved by AP‐HP's Scientific and Ethics Committee (IRB00011591) (approval CSE 20‐0055_COVONCO‐AP) on May 15, 2020. This study conforms to the Declaration of Helsinki standards. French regulations do not require written patient consent for this type of research. In accordance with the European General Data Protection Regulation, patients were informed and those who objected to the secondary use of their data for research were excluded from the study.

## 3 RESULTS

### 3.1 Patients' characteristics (Table 1)

**TABLE 1:** Characteristics of the study population.

	2019	2020
Number of newly referred female BC patients who underwent anticancer treatment at AP‐HP	2055	1988
Patients age median (IQR)	60.5 (49.9–71.5)	61.0 (49.8–71.9)
Anticancer treatment strategy		
• BC resection–no (%)	1499 (72.9)	1425 (71.7)
• Exclusive systemic treatment–no (%)	268 (13.0)	276 (13.9)
• Radiation therapy–no (%)	177 (8.6)	180 (9.1)
• Exclusive best supportive care–no (%)	111 (5.4)	107 (5.4)
Number of hospital SARS CoV‐2 infection diagnoses	22	104

Abbreviations: AP‐HP, Greater Paris Teaching Hospital (Assistance Publique–Hôpitaux de Paris); BC, breast cancer; interquartile, IQR, interquartile range.

**FIGURE 1:**
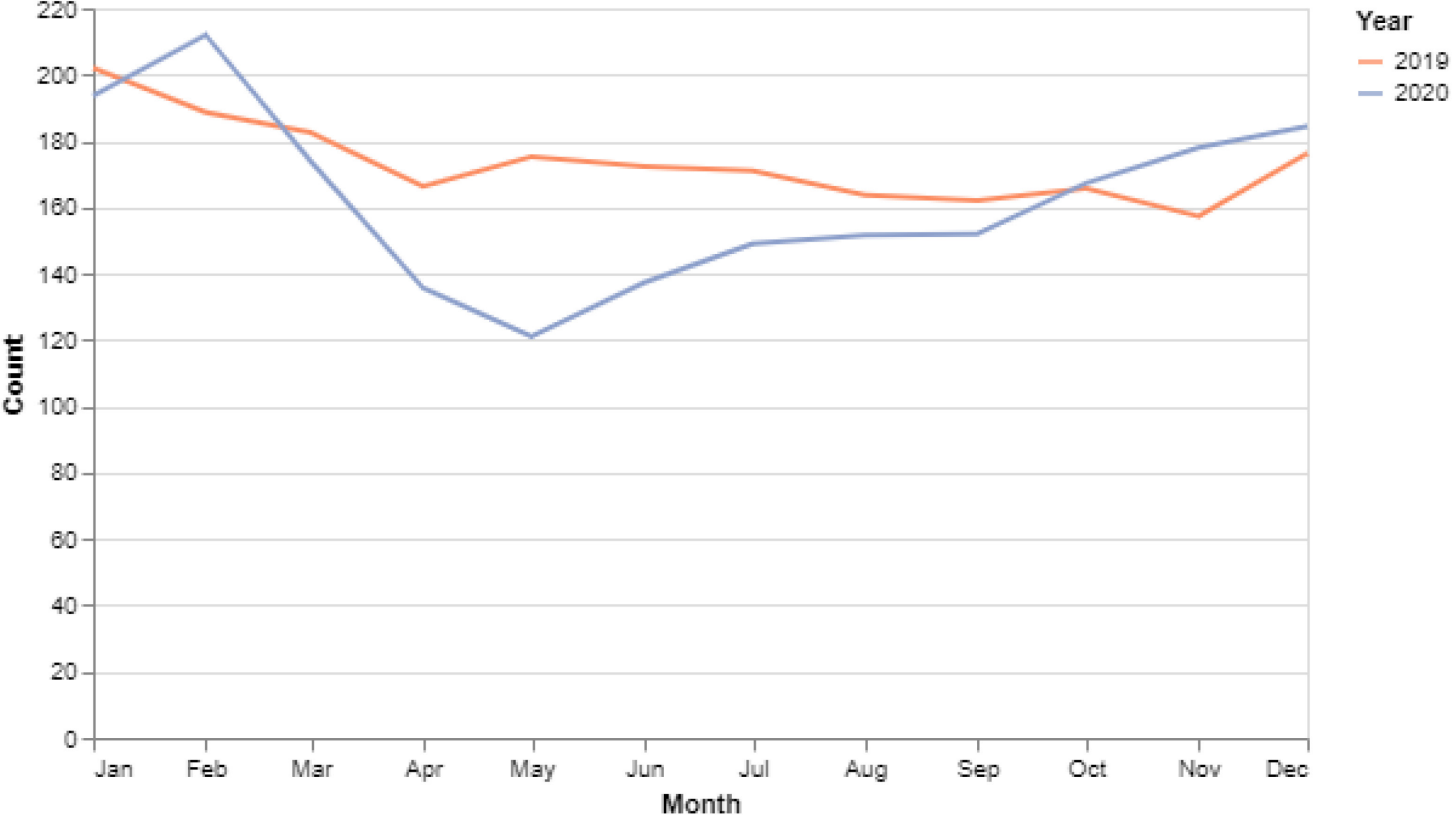
Monthly (3‐month moving average) number of breast cancer cases newly referred to Assistance Publique–Hôpitaux de Paris (AP‐HP) hospitals between January 2019 and December 2020.

Two thousand three hundred and three female patients were newly referred to AP‐HP with a BC diagnosis in 2019, and 2258 in 2020. Patients' median age was 60.5 (interquartile range (IQR): 49.9–71.5) in 2019 and 61.0 (IQR: 49.8–71.9) in 2020 (*p* = 0.62). The number of newly referred BC patients decreased by 18% during the first national lockdown (March–May 2020) and increased by 23% during the second one (October–November 2020) compared to the same periods in 2019 (Figure 1). The number of newly referred elderly patients changed by the same proportions, and we did not find a correlation between the number of newly referred patients and patients' age (Table S2 and Figure S1).

### 3.2 Anticancer treatment strategies

Two thousand and fifty‐five patients received BC treatment in 2019 and 1988 in 2020, respectively (Table 1). One thousand four hundred and ninety‐nine (73%) underwent BC resection in 2019, versus 1425 (72%) in 2020. Two hundred and sixty‐eight patients (13%) had exclusive systemic anticancer therapy in 2019, versus 276 (14%) in 2020. One hundred and seventy‐seven patients (9%) had exclusive radiation treatment in 2019, and 180 (9%) in 2020. Finally, 111 patients (5%) had exclusive BSC in 2019, versus 107 (5%) in 2020. There was no difference in the distribution of patients between treatment categories either overall (*p* = 0.81) or by age categories (Table S3 and Figure S2) (*p* = 0.95).

Among the patients who underwent BC resection, 133 (9%) had a subsequent BC resection in 2019 compared to 141 (10%) in 2020 (*p* = 0.38), 245 (16%) underwent neoadjuvant anticancer treatment in 2019 compared to 283 (20%) in 2020 (*p* = 0.02), 402 (27%) underwent adjuvant chemotherapy in 2019 compared to 355 (25%) in 2020 (*p* = 0.26), and 651 (43%) underwent adjuvant radiation therapy in 2019 compared to 686 (48%) in 2020 (*p* = 0.01) (Tables S4 and S5). In the 50–70 years age group, 108 (15%) patients underwent neoadjuvant treatment in 2019, compared to 137 (21%) in 2020 (*p* = 0.01) (Table S4). There was no significant difference in the age distribution of patients who underwent resection (*p* = 0.65) (Table S4). Two hundred and twenty‐four (15%) patients had reconstructive breast plastic surgery in 2019, compared to 248 (17%) in 2020 (*p* = 0.08). Among patients who underwent total mastectomy, 65/522 (13%) had immediate breast reconstruction in 2019, compared to 93/489 (19%) in 2020, with a rate of breast prosthesis placement reaching 43% and 53% (*p* = 0.30), respectively. Among patients who underwent BC resection, 77 (4%) and 73 (4%) had oncoplastic surgery (of which 58 and 60 occurred during the BC resection) in 2019 and in 2020, respectively (*p* = 0.41).

### 3.3 Care pathways

Among the 4043 patients with any type of anticancer treatment at AP‐HP in 2019 and 2020, 2071 (50%) had a cancer diagnosis performed in AP‐HP teaching hospitals, with a median delay of 35 days (IQR 22–65) between the pre‐treatment pathology report and the initiation of the first treatment. The proportion of patients with a pre‐treatment pathology report and the delay between the report and the initiation of treatment did not vary over time (Figures S3 and S4). Three thousand and forty‐one of the 3831 patients who underwent active anticancer treatment (79%) had an MDM report available. The median time between the first MDM and the first therapeutic procedure was stable at 19 days (IQR 11–39 days) in 2019 and 2020, including during the lockdown periods.

### 3.4 Initial tumor stage

Among the 2924 patients who underwent BC resection, 2762 (94%) had available pathology reports following BC resection. One thousand six hundred and twenty‐eight (59%) of these reports mentioned a (y)pTNM score. In the 1332 patients with upfront BC resection and available data, the repartition between the pTNM risk groups did not change over time: 168 (25%) and 148 (23%) for the high‐risk category in 2019 and 2020, respectively (*p* = 0.37). Among the 296 patients with neo‐adjuvant treatment, 55 (37%) were in the high‐risk ypTNM category in 2019, and 67 (45%) in 2020 (*p* = 0.31). Among the 725 (17.9% of the overall population) patients with an available baseline PET‐scan and/or a CT‐scan report, the proportion of metastatic cancers did not differ over time: 15% and 15% in 2019 and 2020, respectively (Figures S5–S8 and Tables S6 and S7).

### 3.5 Clinical outcomes and overall survival

**FIGURE 2:**
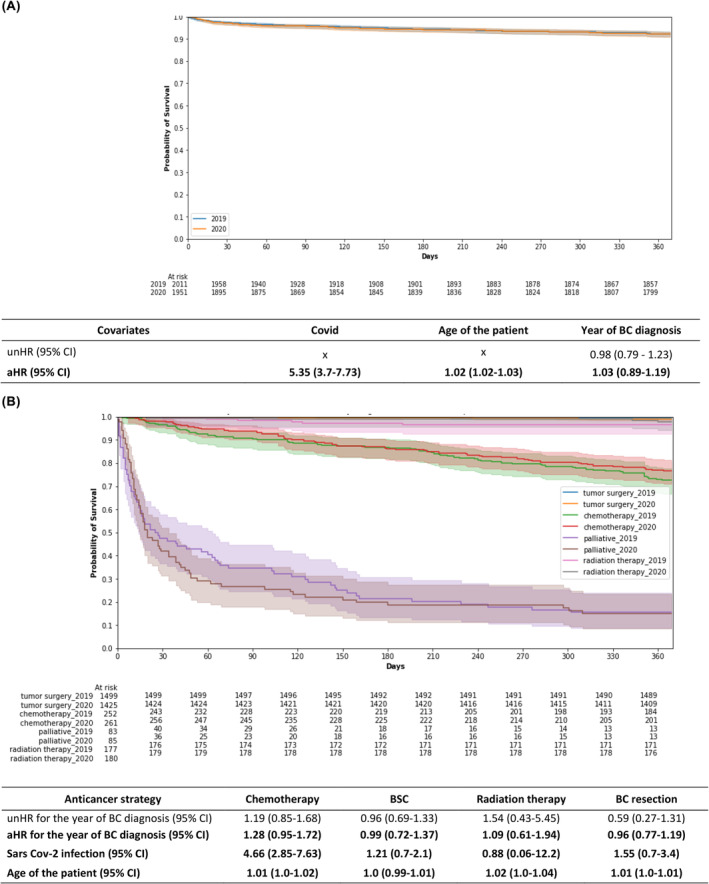
One‐year OS of newly referred breast cancer patients in (A) the overall population, and in patients who underwent (B) upfront tumor resection, exclusive anticancer systemic therapy, exclusive radiation therapy, or best supportive care in 2019 and 2020 in AP‐HP teaching hospitals. aHR, adjusted hazard ratio; AP‐HP, Assistance Publique–Hôpitaux de Paris; BC, breast cancer; BSC, best supportive care; CI, confidence interval; OS; overall survival; unHR, unadjusted H.

In the overall population, the 1‐year OS rate did not differ between 2019 and 2020 when patients' age and the occurrence of a SARS CoV‐2 infection were taken into consideration (92.3% vs. 92.1% [HR = 1.0; 95% CI: 0.89–1.20]) (Figure 2A). The OS rate remained stable between 2019 and 2020 across all treatment categories: patients undergoing tumor resection (99.3% vs. 98.9% in 2019 and 2020, respectively [HR = 1.0, 95% CI: 0.8–1.2]), exclusive systemic anticancer therapy (72.6% vs. 76.6% [HR = 1.3, 95% CI: 1.0–1.7]), exclusive radiation therapy (96.6% vs. 97.8% [HR = 1.09; 95% CI: 0.61–1.94]), or BSC (15.5% vs. 15.1% [HR = 1.0; 95% CI, 0.7–1.4]) (Figure 2B). Older age was associated with a lower 1‐year OS, both in the overall population and in individual treatment categories (Figure 2B).

### 3.6 SARS CoV‐2 infection

In 2020, 48 patients undergoing tumor resection had a proven SARS CoV‐2 infection (3% of patients with BC resection that year), 33 (11%) of those with exclusive systemic anticancer therapy, 4 (2%) of those with exclusive radiation therapy, and 19 (17%) of those who received BSC. Patients with SARS CoV‐2 infection had a significantly lower OS rates than patients without infection in the overall population (HR = 5.3, [95% CI: 3.7–7.7]) (Figure 2B), and in patients with exclusive systemic anticancer therapy (HR = 4.7 [95% CI: 2.9–7.6]), but not for patients undergoing tumor resection (HR = 1.5 [95% CI: 0.7–3.4]), exclusive radiation therapy (HR = 0.9 [95% CI: 0.1–12.0]) or BSC (HR = 1.2 [95% CI: 0.7–2.1]) (Figure 2B).

## 4 DISCUSSION

In this large multicenter cohort study using real‐life data, we observed a significant decrease in the number of patients newly referred for BC to AP‐HP hospitals during the first national SARS CoV‐2 lockdown, with a subsequent catch‐up. The timeline of initial hospital management, the distribution of tumor stages, the repartition of patients across treatment categories (including plastic surgery), and the 1‐year OS rate did not differ significantly between 2019 and 2020. Older patients were represented in all subpopulations with no significant differences compared to younger patients. However, older age was associated with poorer clinical outcomes, regardless of the occurrence of a SARS CoV‐2 infection or the type of BC cancer treatment strategy.

Other international studies showed a decrease in the number of newly referred BC patients during the beginning of the pandemic, mostly due to the interruption of national programs of BC screening.^4^, ^5^, ^6^, ^29^, ^30^ In France, the National Cancer Institute (Institut national du cancer, INCa) stopped the national BC screening program between March and June 2020, following scientific guidelines.^17^, ^31^ Yet, in this study, the overall number of new BC cases diagnosed in 2020 was not different from the 2019 figures, which indicates that patients not seen during the lockdown were seen later in the year. Moreover, the age distribution of patients was stable throughout the study period. In Paris area, dedicated BC screening programs were launched after the initial outbreak to catch up the potential BC diagnosis delays efficiently, according to national guidelines.^32^, ^33^ In England, the screening program resumed in July 2020 and a recent study showed figures consistent with ours. After a 9% decrease in 2020, as many new BC referrals were observed in 2021 as pre‐pandemic, but with a larger proportion of “urgent referrals.”^7^ A Spanish study showed that BC was over‐diagnosed by 14% in 2020–2021 compared to the pre‐pandemic period.^34^ In other countries such as the US, the number of diagnoses has not yet recovered its pre‐pandemic level.^8^ An American study based on the 1,600,000 annual BC screening mammography performed between 2017 and 2022 showed that the rates of mammography performed between March 2020 and February 2021, and between March 2021 and April 2022, were 17% and 4% lower than expected, respectively.^35^ This decrease in BC screenings may have exacerbated preexisting social disparities.^36^

Our study showed that hospital delays between diagnostic breast biopsies, first MDM, and first administration of cancer treatment did not vary over time. These results highlight that hospital BC patients were managed as usual during the initial waves of the pandemic, as other BC centers have reported in Italy.^37^ This finding is at odds with the results of a quasi‐experimental study performed in six European countries, which found that the median time‐to‐treatment for newly diagnosed BC patients in 2020 may have increased compared to the 2017–2019 period, with significant heterogeneity between countries.^38^ Other studies reported comparable results, with a negative impact on patient‐reported outcomes.^39^, ^40^, ^41^, ^42^

We observed no significant impairment in the initial clinical presentation or the anticancer therapeutic strategies during and after the SARS CoV‐2 outbreak. This is also true for surgeries, despite pressures on anesthesia during the pandemic. Patients did not present with a higher tumor burden due to delays in BC diagnostic procedures or access to anticancer therapeutic strategies, independently from their age. In the patient group aged from 50 to 70 years, the administration of neoadjuvant chemotherapy may have increased in 2020 compared to 2019. Yet, there were no differences in ypTNM and pTNM in 2020 compared to 2019 for patients with neoadjuvant chemotherapy and upfront BC resection, respectively. This increase in neoadjuvant chemotherapy in 2020 may be due to logistic care pathway issues rather than to delays in BC diagnoses or to more advanced tumors at initial presentation.^43^ Modified strategies for localized BC care were observed in other developed countries during the outbreak. This approach may have helped to maintain the time‐to‐first‐treatment stable and comparable to the pre‐pandemic level during the outbreak.^44^

Our results are at odds with other studies that found a shift in initial tumor staging in new BC cases following the pandemic. A Japanese study found increased rates of stage IIB or more after the pandemic outbreak compared to 2019.^10^ An English study on 439 patients showed more advanced BCs at initial presentation, with higher rates of node‐positive and metastatic stages in 2020 compared to 2019.^45^ Other studies in the US, South America, Europe, the Middle East, and Asia, obtained similar results.^12^, ^13^, ^14^, ^30^, ^43^, ^46^, ^47^, ^48^, ^49^ Studies showing no tumor shift, as displayed in the presented results, are scarce,^11^, ^15^ but may also have been underreported. Our study showed no difference in terms of frequency and modalities of breast reconstruction surgery in 2020 compared to 2019, unlike other studies that found less breast reconstruction surgeries post‐pandemic, in consistence with scientific recommendations.^15^, ^50^, ^51^, ^52^, ^53^

After adjustment on patients' age and occurrence of a SARS CoV‐2 infection, the mortality rate of patients in this study patients was not different in 2020, compared to 2019, across treatment categories. Older age was an independent negative prognostic factor, regardless of the therapeutic strategy.^54^ As expected, the occurrence of a SARS CoV‐2 infection was associated with a fourfold increase in mortality risk in patients treated with exclusive chemotherapy.^55^

Among the study strengths, we report one of the largest published cohorts of newly referred BC patients with one‐year clinical outcomes, in an area severely hit by the SARS CoV‐2 outbreak. Our analysis relies on extensive analysis of EHR data, from which we assessed their care management, initial clinical presentation, and 1‐year clinical outcomes. We identified BC cases and therapeutic strategies based on hospital claims. Cancer registries remain the gold standard for such analyses, but studies have concluded that French claims databases are reliable for our scientific purpose.^56^ Among the study limits, our results are limited to one regional healthcare provider, despite the size of the database we investigated (11 million patient records). This generates limitations. For example, only 9% of BC patients in our study received exclusive radiation therapy. Their OS rate suggests that at least some of them had BC resection elsewhere, highlighting the fact that we did not have access to data on patient care outside of this provider, such as most of BC diagnostic biopsies and initial screening mammograms. Even in our database, data availability was an issue for some items, for example, for TNM scores. In parallel, the outbreak may have modified hospital recruitment patterns, biasing comparisons between 2019 and 2020. The natural language processing algorithms we used to identify tumor stages from EHRs may lack classification precision. Finally, the occurrence of SARS CoV‐2 infection may have been underestimated as the use of diagnostic tests was not systematic at the beginning of the outbreak.

## 5 CONCLUSION

Despite a significant decrease in the number of patients newly referred for a BC during the first pandemic wave, the tumor stages, the initial hospital delays of care management, the distribution across therapeutic strategy categories, and the 1‐year OS rate did not vary significantly between 2019 and 2020. Prognostic factors such as the occurrence of a SARS CoV‐2 infection during chemotherapy course or older age were confirmed. Larger population‐based studies with longer follow‐up will be necessary to assess the long‐term impact of the SARS CoV‐2 outbreak on newly referred BC patients.

## AUTHOR CONTRIBUTIONS

**Etienne Guével:** Data curation (lead); formal analysis (supporting); methodology (supporting); software (equal); validation (lead); visualization (lead); writing – review and editing (supporting). **Sonia Priou:** Conceptualization (lead); data curation (lead); formal analysis (lead); investigation (equal); methodology (lead); software (lead); validation (lead); visualization (equal); writing – review and editing (lead). **Guillaume Lamé:** Conceptualization (lead); data curation (supporting); formal analysis (lead); funding acquisition (lead); investigation (lead); methodology (lead); project administration (supporting); resources (supporting); supervision (lead); validation (lead); visualization (lead); writing – original draft (lead); writing – review and editing (lead). **Johanna Wassermann:** Investigation (equal); validation (equal); writing – review and editing (lead). **Romain Bey:** Conceptualization (supporting); data curation (supporting); funding acquisition (supporting); investigation (equal); methodology (supporting); project administration (supporting); resources (supporting); software (supporting); supervision (supporting); visualization (supporting); writing – review and editing (supporting). **Catherine Uzan:** Formal analysis (supporting); resources (supporting); validation (supporting); writing – review and editing (supporting). **Gilles Chatellier:** Conceptualization (lead); data curation (supporting); formal analysis (lead); investigation (lead); methodology (lead); supervision (lead); validation (lead); visualization (lead); writing – original draft (supporting); writing – review and editing (lead). **Yazid Belkacemi:** Formal analysis (equal); investigation (equal); methodology (equal); resources (equal); validation (equal); writing – review and editing (lead). **Xavier Tannier:** Conceptualization (lead); data curation (supporting); formal analysis (lead); investigation (lead); methodology (lead); resources (equal); software (lead); supervision (lead); validation (lead); visualization (equal); writing – original draft (supporting); writing – review and editing (lead). **Sophie Guillerm:** Formal analysis (equal); investigation (equal); methodology (equal); resources (equal); validation (lead); writing – review and editing (lead). **Rémi Flicoteaux:** Conceptualization (supporting); data curation (equal); formal analysis (supporting); investigation (equal); methodology (equal); supervision (equal); validation (lead); visualization (equal); writing – original draft (supporting); writing – review and editing (lead). **Joseph Gligorov:** Conceptualization (equal); formal analysis (equal); investigation (equal); methodology (equal); resources (equal); supervision (equal); validation (lead); writing – original draft (supporting); writing – review and editing (lead). **Ariel Cohen:** Data curation (lead); formal analysis (lead); investigation (lead); methodology (lead); resources (supporting); software (lead); validation (lead); visualization (lead); writing – original draft (supporting); writing – review and editing (lead). **Marc‐Antoine Benderra:** Formal analysis (equal); investigation (equal); methodology (equal); resources (equal); validation (equal); writing – original draft (supporting); writing – review and editing (lead). **Luis Teixeira:** Investigation (equal); methodology (equal); resources (equal); validation (equal); writing – review and editing (lead). **Christel Daniel:** Conceptualization (equal); data curation (equal); formal analysis (equal); funding acquisition (equal); investigation (equal); methodology (equal); project administration (lead); resources (lead); software (lead); supervision (supporting); validation (equal); visualization (equal); writing – review and editing (lead). **Barbara Hersant:** Conceptualization (lead); formal analysis (equal); investigation (equal); methodology (equal); supervision (equal); validation (equal); writing – review and editing (lead). **Christophe Tournigand:** Conceptualization (equal); formal analysis (equal); funding acquisition (lead); investigation (lead); methodology (equal); project administration (lead); resources (lead); supervision (lead); validation (supporting); writing – original draft (supporting); writing – review and editing (lead). **Emmanuelle Kempf:** Conceptualization (lead); data curation (lead); formal analysis (lead); funding acquisition (lead); investigation (lead); methodology (lead); project administration (lead); resources (lead); software (supporting); supervision (lead); validation (lead); visualization (lead); writing – original draft (lead); writing – review and editing (lead).

## FUNDING INFORMATION

This research was supported by a grant from the AP‐HP foundation and by a grant from ARC Foundation for cancer research (grant reference COVID202001343).

## CONFLICT OF INTEREST STATEMENT

The authors have no conflict of interest to disclose.

## ETHICS STATEMENT

The French Data Protection Agency (CNIL, Commission Nationale de l'Informatique et des Libertés) authorized the constitution of the AP‐HP Clinical Data Warehouse (CDW) on January 19, 2017 (approval no. 19800120). In a single commitment to the CNIL, AP‐HP declared complying with the French national reference methodology MR‐004 (https://www.legifrance.gouv.fr/jorf/id/JORFTEXT000037187498) governing the processing of personal data for the purposes of research of a public interest nature that does not involve the human person, in particular research reusing previously registered data. Finally, the present research was approved by the AP‐HP Scientific and Ethics Committee (IRB00011591) (approval CSE 20‐0055_COVONCO‐AP) on May 15, 2020. This study conforms to the Declaration of Helsinki standards. French regulations do not require written patient consent for this type of research. In accordance with the European General Data Protection Regulation, patients were informed and those who objected to the secondary use of their data for research were excluded from the study.

## Supporting information


Data S1:

## Data Availability

The data and the code that support the findings of this study are available on request from the corresponding author. The process to access the raw data of the clinical data warehouse is described on its website eds.aphp.fr.
