# Supplementary material for: Impact of the COVID‐19 pandemic on clinical presentation, treatments, and outcomes of new breast cancer patients: A retrospective multicenter cohort study
Source: Cancer Med. 2023 Nov 1;12(22):20918–29. doi: 10.1002/cam4.6637 (PMC10709737; doi:10.1002/cam4.6637)
Supplement: Supplementary file 1 — Data S1: [file CAM4-12-20918-s001.docx]

**Supplementary data**

**Supplementary Methods**

**Supp Table 1.** Diagnosis and treatment codes related to the 4 categories of anticancer treatment strategies.

- Breast biopsy:

QEHB001, QEHA002, QEHJ005, QEHJ001, QEHH001, QEHJ006, QEHB002, QEHH003, QEHH015, QEHH002, QEHJ004, QEHJ002, QEHJ003, QEQX016, QEQX037, QEHA001 CCAM codes

- Breast cancer surgery:

QEFA018, QEFA001, QEFA020, QEFA004, QEFA019, QEFA017, QEFA008, QEFA007, QEFA003, QEFA016, QEFA010, QEFA005, QEFA013, QEFA012, QEFA015 CCAM codes

- Exclusive systemic anticancer therapy:

ICD10 code Z511

- Exclusive radiation therapy:

ICD10 code Z5101

- Exclusive best supportive care :

ICD10 code Z515

Abbreviations: CCAM, Common Classification of Medical Procedures; ICD10, international code diseases 10th edition

**Description of the development and validation of the NLP techniques used to extract the metastatic status at initial presentation of BC cases.**

The **metastatic status of BC cases** at initial presentation has been automatically extracted from the report of PET/CT-scans and of CT-scans performed between 90 days before and 45 days after the BC diagnosis date using a regular expression algorithm. Baseline CT-scan identification methodology has been previously published ^27^.

**PET/CT-scans identification**

The PET/CT-scans were identified with a dedicated algorithm based on regular expression rules.

PET/CT-scan examinations were identified and then defined as likely related to a baseline evaluation according to the procedure, as follows:

- PET/CT-scan:
  - Text containing the following French expression concerning fluorodeoxyglucose18 (product commonly used for the imaging contrast in PET/CT-scan) : **\b(((18)?f-fdg)|(flu(oro)?désoxyglucose)|(fdg-\b18f\b))**
  - Text containing the combination of both French expressions concerning PET/CT-scan : **\b((tep)|(pet)|(tomo(scinti)?graphie par [ée]mission de position(s)?))(\s|-|\/)**

and nuclear activity :

**\b((m[ée]decine nucl[ée]aire)|([0-9]{1,4}((\s)?)mbq)|([Aa]ctivité inject[eé](e)?))**

- Procedure performed between 90 days before and 45 days after the BC diagnosis date

**Dataset**

Overall, 280 PET/CT-scan text reports were manually annotated by a senior medical oncologist. The annotator was asked to classify the report as staging or non-staging PET/CT-scan, and to specify the metastatic status of the related tumor. This annotated dataset was used for the development of both algorithms and randomly split into a training set (182 reports, 65%) and a validation set (98 reports, 35%).

**Metastatic status**

We extracted the metastatic status of BC cases at initial presentation from the available imaging text reports within the electronic health record (EHR) of each patient using machine learning algorithms with two sequential steps.

To that aim, we identified the PET/CT-scans within the imaging text reports available in the CDW according to the above-described algorithm. We selected the first hospital PET/CT-scan performed between 90 days before and 45 days after the BC diagnosis date. We then compared three methods of binary classification to distinguish staging PET/CT-scans from non-staging PET/CT-scans. All methods are based on machine learning:

1. A random forest algorithm based on the frequency of words from the medical history and the conclusion sections of the text reports,
2. A gradient boosting classifier based on the frequency of words from the medical history and the conclusion sections of the text reports,
3. A convolutional neural network (CNN) using word2vec word embeddings pretrained on the CDW HER

The random forest tree and the gradient boosting classifier models were taken from the library scikit-learn (v1.0.2) . torch (v1.12.0) was used to develop and train the CNN model.

A portion of the dataset was used as a development dataset (55 CRs, 30% of the training dataset) to:

- Perform a gridsearch to find the best hyperparameters in the case of the gradient boosting classifier and the random forest classifier
- Evaluate the accuracy and loss of the CNN classifier at every epoch, and decide when to stop the training

The metastatic status of BC cases at initial presentation was extracted using the following regular expression algorithm on the training set solely:

(?i)(m[ée]tasta(se|tique)s?)|(diss[ée]min([ée]|ation))|(carcinose)|(((allure|l[ée]sion|localisation|progression)s?\s)(suspecte?s?)?[^\.\!\?]{0,50}(secondaire)s?)|(l(a|â)ch(é|e|er)\sde\sballons?)|(l[ée]sions?\s(non\s)?cibles?)|(rupture[^\.\!\?]{1,20}corticale)|(envahissement[^\.\!\?]{0,15}parties\smolles)|(\s(l[i,y]se)[^\.\!\?]{1,20}os)|ost[eé]ol[i,y]|rupture[^\.\!\?]{1,20}corticale|envahissement[^\.\!\?]{1,20}parties\smolles|ost[eé]ocondensa[^\.\!\?]{1,20}(suspect|secondaire|[ée]volutive)|(l[ée]sion|anomalie|image)[^\.\!\?]{1,20}os[^\.\!\?]{1,30}(suspect|secondaire|[ée]volutive)|os[^\.\!\?]{1,30}(l[ée]sion|anomalie|image)[^\.\!\?]{1,20}(suspect|secondaire|[ée]volutive)|(l[ée]sion|anomalie|image)[^\.\!\?]{1,20}l[i,y]tique|(l[ée]sion|anomalie|image)[^\.\!\?]{1,20}condensant[^\.\!\?]{1,20}(suspect|secondaire|[ée]volutive)|fracture[^\.\!\?]{1,30}(suspect|secondaire|[ée]volutive)|((l[ée]sion|anomalie|image|nodule|atteinte)[^\.\!\?]{1,80}(secondaire))|((l[ée]sion|anomalie|image|nodule)s[^\.\!\?]{1,40}suspec?ts?)

The strings matched by the previous regex which were detected as negations or hypotheses were eliminated. The qualification of negation of hypothesis were made using the edsnlp library (v0.7.4)

For both algorithms, on the training and the validation sets, the following performance metrics were evaluated: sensitivity (recall), predictive positive value (precision), f1 score (harmonic mean between sensitivity and predictive positive value).

**Supplementary Results**

**Supplementary Table 2**. Number of new breast cancer diagnoses across age category

ies, according to the month of breast cancer diagnosis

| **Patients’ age** | **< 50 years** | | | | **50 – 70 years** | | | | **70 – 80 years** | | | **> 80 years** | | |
| --- | --- | --- | --- | --- | --- | --- | --- | --- | --- | --- | --- | --- | --- | --- |
| **Year of BC diagnosis** | **2019** | | **2020** | **% difference**  **2020 versus 2019** | **2019** | **2020** | **% difference**  **2020 versus 2019** | | **2019** | **2020** | **% difference**  **2020 versus 2019** | **2019** | **2020** | **% difference**  **2020 versus 2019** |
| Month of BC diagnosis |  | | | | | | |  |  |  |  |  |  |  |
| - January | 50 | | 66 | +32% | 92 | 101 | +10% | | 38 | 43 | +12% | 24 | 19 | -21% |
| - February | 52 | | 45 | -14% | 95 | 105 | +10% | | 34 | 43 | +26% | 19 | 17 | -11% |
| - March | 41 | | 48 | +17% | 72 | 81 | +12% | | 33 | 37 | +12% | 16 | 31 | +93% |
| - April | 47 | | 32 | -32% | 80 | 54 | -32% | | 33 | 18 | -46% | 26 | 11 | -58% |
| - May | 39 | | 29 | -26% | 67 | 37 | -45% | | 21 | 15 | -29% | 24 | 14 | -42% |
| - June | 41 | | 41 | 0 | 92 | 65 | -29% | | 37 | 21 | -43% | 19 | 26 | +37% |
| - July | 47 | | 48 | +2% | 89 | 69 | -23% | | 29 | 28 | -4% | 12 | 19 | +58% |
| - August | 27 | | 30 | +11% | 78 | 54 | -31% | | 30 | 33 | +10% | 12 | 13 | +8% |
| - September | 55 | | 37 | -33% | 80 | 65 | -19% | | 17 | 39 | +129% | 15 | 20 | +33% |
| - October | 52 | | 43 | -17% | 67 | 90 | +34% | | 32 | 19 | -41% | 21 | 13 | -38% |
| - November | 40 | | 45 | +12% | 72 | 84 | +17% | | 34 | 33 | -3% | 12 | 14 | +17% |
| - December | | 33 | 47 | +42% | 70 | 91 | +30% | | 22 | 37 | +68% | 17 | 18 | +6% |

Abbreviation: BC, breast cancer


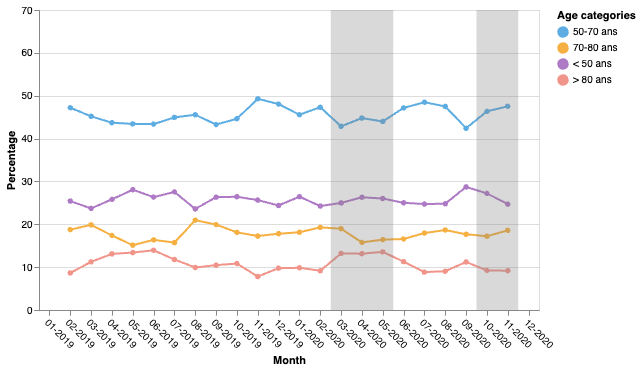

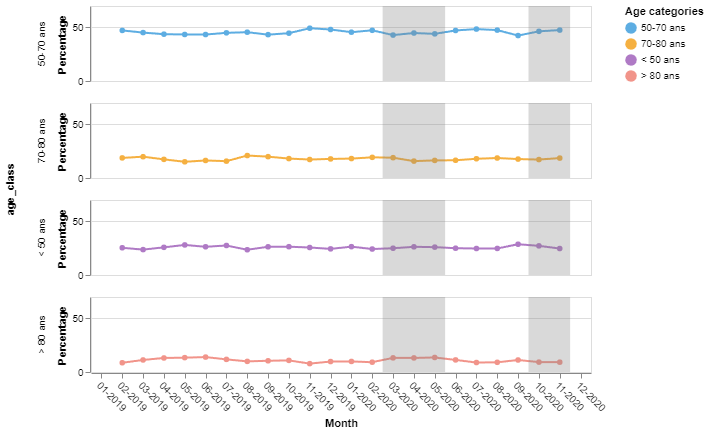


**Supplementary Fig. 1:** 3-month averaged percentage of newly diagnosed breast cancer patients by category of age at the breast cancer diagnosis. Grey areas correspond to national lockdown periods.

**Supplementary Table 3**. Distribution of anticancer treatment strategies across age categories, according to the year of breast cancer diagnosis

| **Age category** | **< 50 years** | | **50-70 years** | | **70-80 years** | | **> 80 years** | |
| --- | --- | --- | --- | --- | --- | --- | --- | --- |
| **Year of BC diagnosis** | **2019** | **2020** | **2019** | **2020** | **2019** | **2020** | **2019** | **2020** |
| **Total number of patients** | 524 | 511 | 954 | 896 | 360 | 366 | 217 | 215 |
| - **BC resection** | 418 (79.8 %) | 400 (78.3 %) | 716 (75.1 %) | 668 (74.6 %) | 245 (68.1 %) | 254 (69.4 %) | 120 (55.3 %) | 103 (47.9 %) |
| - **Exclusive systemic anticancer treatment** | 60 (11.5 %) | 63 (12.3 %) | 132 (13.8 %) | 126 (14.1 %) | 48 (13.3 %) | 50 (13.7 %) | 28 (12.9 %) | 37 (17.2 %) |
| - **Exclusive radiation therapy** | 35 (6.7 %) | 40 (7.8 %) | 80 (8.4 %) | 84 (9.4 %) | 42 (11.7 %) | 35 (9.6 %) | 20 (9.2 %) | 21 (9.8 %) |
| - **Exclusive best supportive care** | 11 (2.1 %) | 8 (1.6 %) | 26 (2.7 %) | 18 (2.0 %) | 25 (6.9 %) | 27 (7.4 %) | 49 (22.6 %) | 54 (25.1 %) |
| ***p*-value** | 0.77 | | 0.67 | | 0.83 | | 0.42 | |

Abbreviation: BC, Breast Cancer


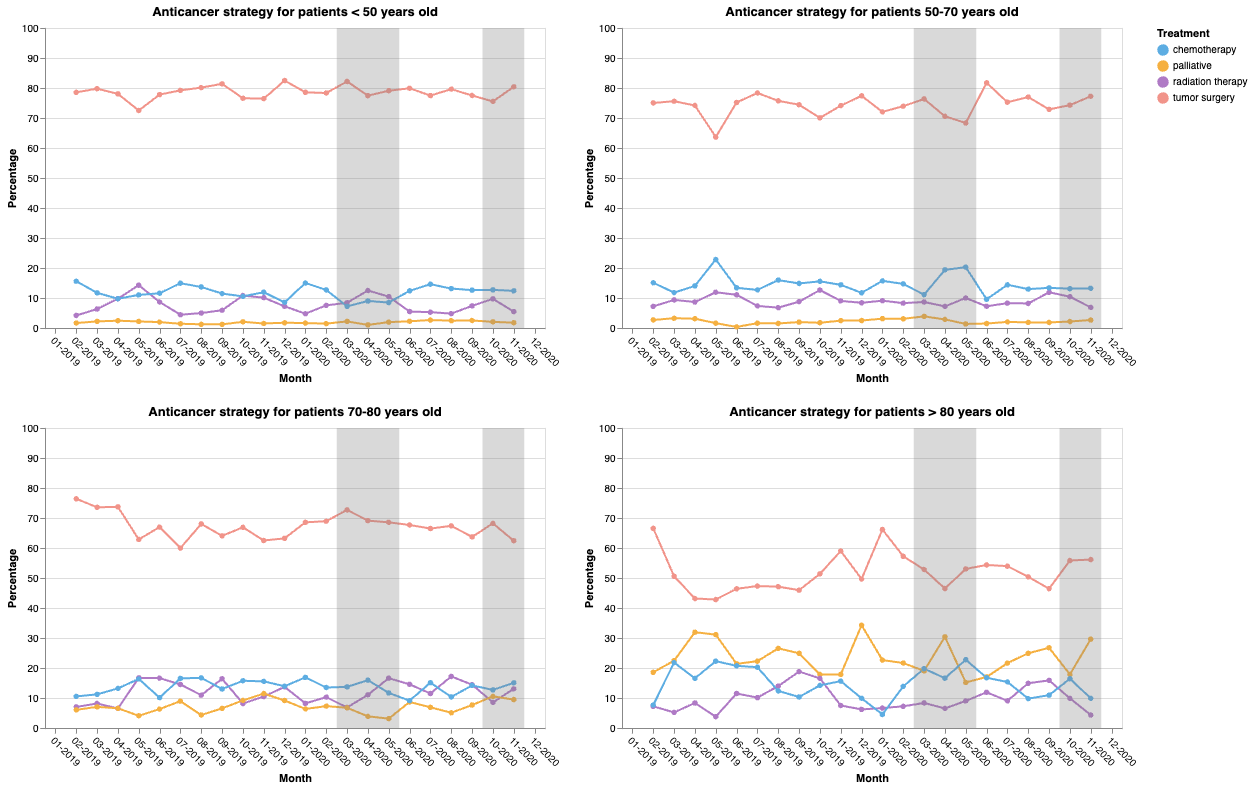


**Supplementary Fig. 2**. Repartition (3-month moving average) of initial upfront treatment strategies for breast cancer cases newly referred to AP-HP hospitals between January 2019 and December 2020, across age categories. In grey, the national lockdown periods of 2020.

**Supplementary Table 4**. Distribution of patients treated with neoadjuvant chemotherapy before breast cancer surgery, across age categories and according to the year of breast cancer diagnosis.

| **Age category** | **< 50 years** | | **50-70 years** | | **70-80 years** | | **> 80 years** | |
| --- | --- | --- | --- | --- | --- | --- | --- | --- |
| **Year of BC diagnosis** | **2019** | **2020** | **2019** | **2020** | **2019** | **2020** | **2019** | **2020** |
| **Patients with BC resection** | 418 | 400 | 716 | 668 | 245 | 254 | 120 | 103 |
| **Neoadjuvant chemotherapy** | 115 (27.5 %) | 120 (30.0 %) | 107 (14.9 %) | 137 (20.5 %) | 19 (7.8 %) | 25 (9.8 %) | 3 (2.5 %) | 1 (1.0 %) |
| ***p*-value 2019 vs 2020** | 0.48 | | 0.01 | | 0.51 | | 0.73 | |

Abbreviation: BC: Breast Cancer

Chi-square test for difference in age distribution between 2019 and 2020: p = 0.65

**Supplementary Table 5.** Distribution of patients treated with adjuvant therapies after breast cancer surgery, across age categories and according to the year of breast cancer diagnosis.

| **Age category** | **< 50 years** | | **50-70 years** | | **70-80 years** | | **> 80 years** | |
| --- | --- | --- | --- | --- | --- | --- | --- | --- |
| **Year of BC diagnosis** | **2019** | **2020** | **2019** | **2020** | **2019** | **2020** | **2019** | **2020** |
| **Patients with BC resection** | 418 | 400 | 716 | 668 | 245 | 254 | 120 | 103 |
| **Adjuvant chemotherapy** | 144 (34.4 %) | 117 (29.2 %) | 182 (25.4 %) | 183 (27.4 %) | 61 (24.9 %) | 44 (17.3 %) | 15 (12.5 %) | 11 (10.7 %) |
| **Adjuvant radiation therapy** | 176 (42.1 %) | 175 (43.8 %) | 334 (46.6 %) | 351 (52.5 %) | 101 (41.2 %) | 126 (49.6 %) | 40 (33.3 %) | 34 (33.0 %) |
| **At least one adjuvant therapeutic procedure** | 250 (59.8 %) | 213 (53.2 %) | 422 (58.9 %) | 425 (63.6 %) | 127 (51.8 %) | 144 (56.7 %) | 46 (38.3 %) | 39 (37.9 %) |
| ***p*-value 2019 vs 2020** | 0.07 | | 0.08 | | 0.32 | | 1.0 | |

Abbreviation: BC: Breast Cancer

Chi-square test for difference in age distribution between 2019 and 2020: p = 0.65


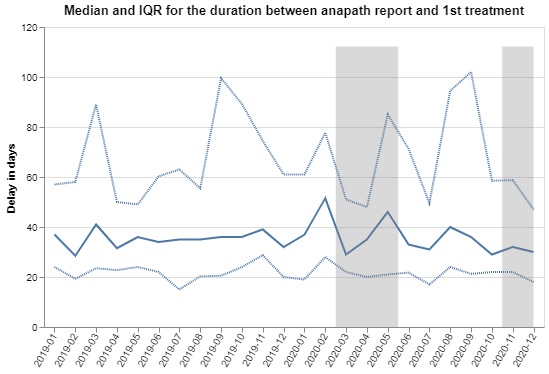


**Supplementary Figure 3.** Median (solid line) and Interquartile range (dotted lines) for the duration between the pathology report and the first cancer treatment (for patients that had both)


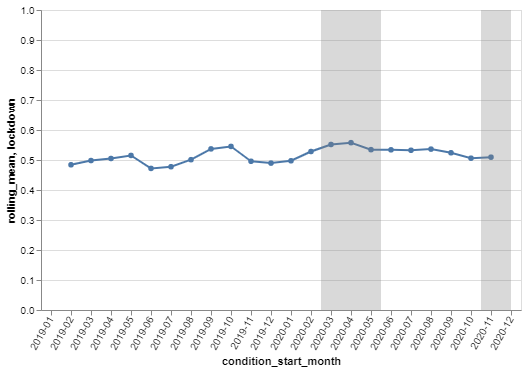


**Supplementary Figure 4**. Rolling average (3 months) of the number of patients that had a pathology report before their first cancer treatment

*Staging PET/CT-scans classification*

The identification algorithm to classify between staging and non-staging PET/CT scans among the initial imaging reports is based on the gradient boosting classifier. It resulted in a sensitivity and a positive predictive value of 99% and 98% on the training set, 98% and 98% on the development set, and 89% and 97% on the validation set respectively.

The identification algorithm based on the random forest tree for staging PET/CT-scans among the initial imaging reports resulted in a sensitivity and a positive predictive value of 99% and 89% on the training set, 100% and 95% on the development set, and 97% and 82% on the validation set respectively.

The identification algorithm based on the CNN for staging PET/CT-scans among the initial imaging reports resulted in a sensitivity and a positive predictive value of 100% and 100% on the training set, 97% and 84% on the development set, and 92% and 86% on the validation set respectively.

**Supp Table 6.** Results of the models on the different datasets

| model | dataset | sensitivity | Positive predictive value | f1 score |
| --- | --- | --- | --- | --- |
| Gradient boosting classifier | training | 0.99 | 0.98 | 0.98 |
|  | development | 0.98 | 0.98 | 0.98 |
|  | validation | 0.89 | 0.97 | 0.93 |
| Random forest classifier | training | 0.99 | 0.89 | 0.94 |
|  | development | 1.00 | 0.95 | 0.98 |
|  | validation | 0.97 | 0.82 | 0.89 |
| CNN | training | 1.00 | 1.00 | 1.00 |
|  | development | 0.97 | 0.84 | 0.90 |
|  | validation | 0.92 | 0.86 | 0.89 |


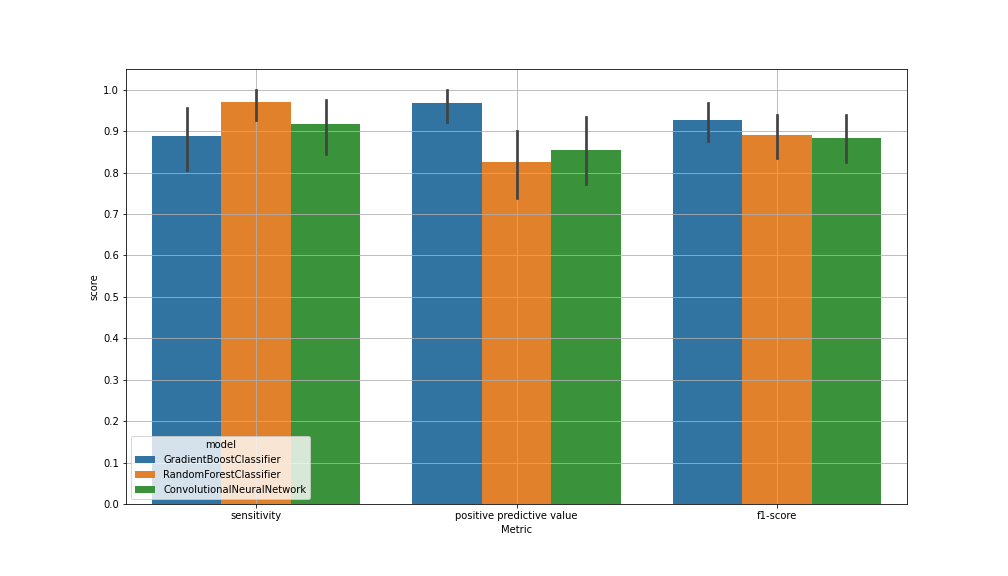


**Supplementary Figure 5.** Comparison of the performances on the validation dataset of the models tested for baseline classification

**Interpretability**

No interpretability is necessary for the algorithms based on regular expressions. Several examination techniques were used to understand the identification algorithm of staging PET/CT-scan. First, SHapley Additive exPlanations (SHAP) summary plot was used to analyse the importance of each word in the classification of documents (Lundberg SM. A unified approach to interpreting model predictions. 2017). The words with the highest weight to classify a document as staging or not staging were “extension” “bilan” “antecedents” “douteux” “connue” and “reste” (Supp Figures 2 and 3). Then, a sensitivity analysis was performed using SHAP force plots. These plots enabled us to understand how words contributed to the model's prediction for a specific document.


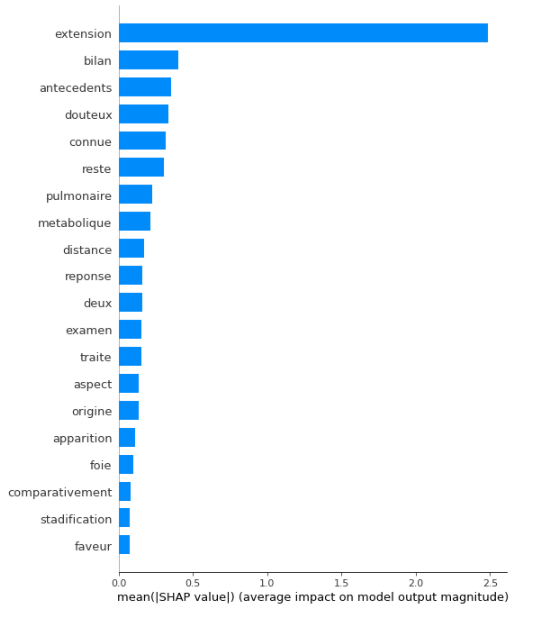


**Supplementary Figure 6**. SHAP summary plot to rank the words used in the PET/CT-scan classification algorithm, according to their respective weights


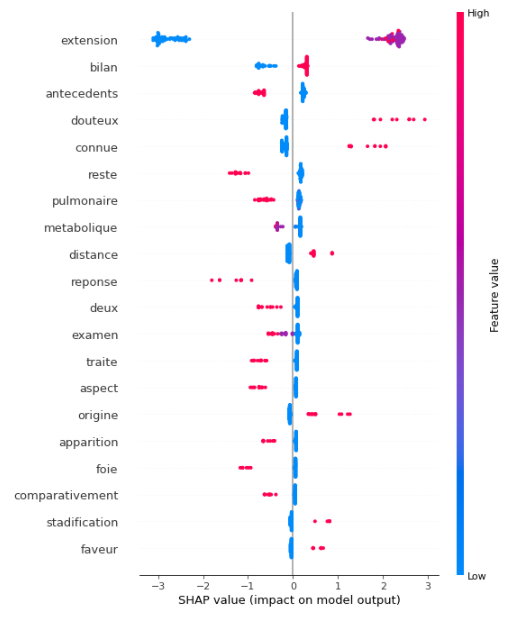


**Supplementary Figure 7**. SHAP summary plot to explain the influence of the most impactful words of the model. Points represent data points, red color if the word is present and the SHAP value the influence of the absence/presence of the word. A positive SHAP value indicate staging and a negative non-staging.

*Metastatic status*

The repartition of the baseline and metastatic reports in both datasets can be found in the Supplementary Table 3.

**Supp. Table 7.** Size and distribution of the different categories inside of the training and validation datasets

|  | Training set | Validation set |
| --- | --- | --- |
| Number of reports | 182 | 98 |
| Number of baseline reports | 125 (69%) | 72 (73%) |
| Number of reports with a metastases mention | 57 (31%) | 23 (23%) |
| Number of baseline reports with a metastase mention | 41 (23%) | 14 (14%) |

The algorithm identified the metastatic status with a sensitivity and a positive predictive value of 74% and 95% on the training set, and 87% and 77% on the validation set respectively. The F1-score of the metastatic algorithm reached 83% and 82% on the training set and on the validation set, respectively.

We also evaluated the performance of the concatenation of the two algorithms (staging of the baseline PET/CT-scan then evaluation of the metastatic status). The concatenation reached a sensitivity and a positive predictive value of 83% and 85% respectively on the training set, and of 79% and 65% on the validation set.

The F1-score of the concatenated algorithms reached 84% and 71% on the training set and on the validation set respectively.


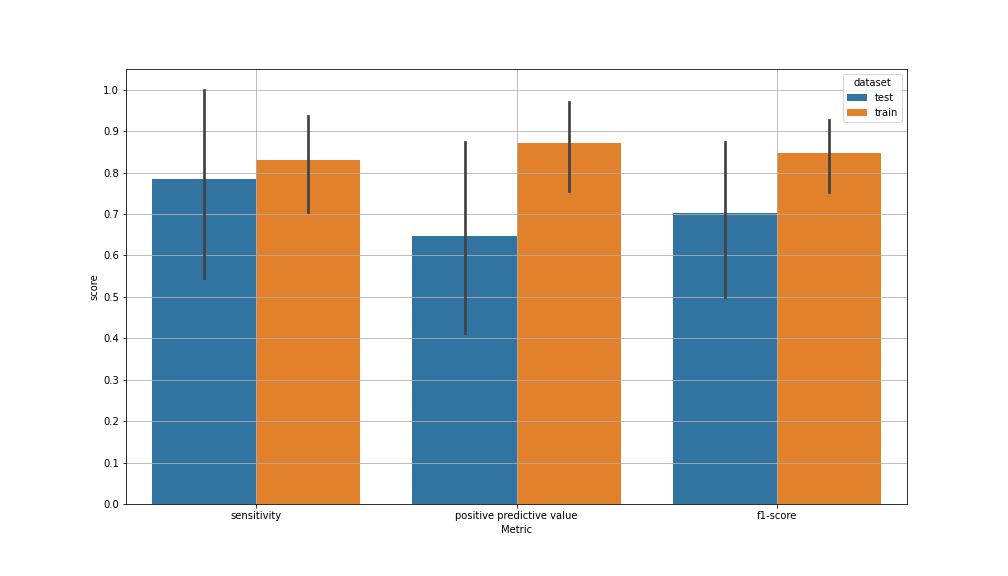


**Supplementary Figure 8**. Comparison of the metrics on the test set for the concatenated algorithms (baseline staging algorithm then metastatic algorithm)
